# Increased ^18^F-FDG uptake of heterotopic pancreatitis in the small intestine: A CARE-compliant case report

**DOI:** 10.1097/MD.0000000000004465

**Published:** 2016-09-09

**Authors:** Maomei Ruan, Min Liu, Lingxiao Cheng, Wenhui Xie, Libo Chen

**Affiliations:** a Department of Nuclear Medicine, Shanghai Jiao Tong University Affiliated Sixth People's Hospital; b Department of Nuclear Medicine, Shanghai Chest Hospital, Shanghai Jiao Tong University, Shanghai, China.

**Keywords:** ^18^F-FDG PET/CT, case report, heterotopic pancreas, pancreatitis, small intestine

## Abstract

**Backgroud:**

Heterotopic pancreas (HP), a relatively uncommon congenital anomaly, is rarely noted during ^18^F-FDG positron-emission tomography/computed tomography (PET/CT) scan.

**Methods:**

A 60-year-old woman was referred to our hospital due to a 10-day history of abdominal pain with elevated levels of serum amylase and lipase. Abdominal CT and ultrasound examinations were negative. In order to search for the cause, an ^18^F-FDG PET/CT whole body scan was suggested to an old woman revealing the presence of ^18^F-FDG accumulating nodule in small intestine.

**Results:**

Surgical findings and pathologic results confirmed the diagnosis of small intestinal heterotopic pancreas with active chronic inflammation.

**Conclusion:**

This uncommon case underscores the necessity of considering heterotopic pancreatitis in small intestine with focal ^18^F-FDG uptake as a possible differential diagnosis in intestinal tumor and tuberculosis.

## 1 Introduction

Heterotopic pancreas (HP) is a relatively uncommon congenital anomaly that is defined as pancreatic tissue without real anatomical or vascular connection to the pancreas.^[1,2]^ Among all abdominal surgeries, the incidence of heterotopic pancreas ranges from 0.25% to 1.2%.^[3,4]^ The most frequent locations are the duodenum (9%–36%), stomach (24%–38%), jejunum (0.5%–27%), and Meckel's diverticulum (2%–6.5%),^[4,5]^ but it can also be found in the ileum, colon, gall bladder, umbilicus, fallopian tube, mediastinum, spleen, and liver.^[3]^ This article reported a case of increased ^18^F-FDG uptake of heterotopic pancreatitis in the small intestine on ^18^F-FDG PET/CT.

## 2 Case report

**Figure 1:**
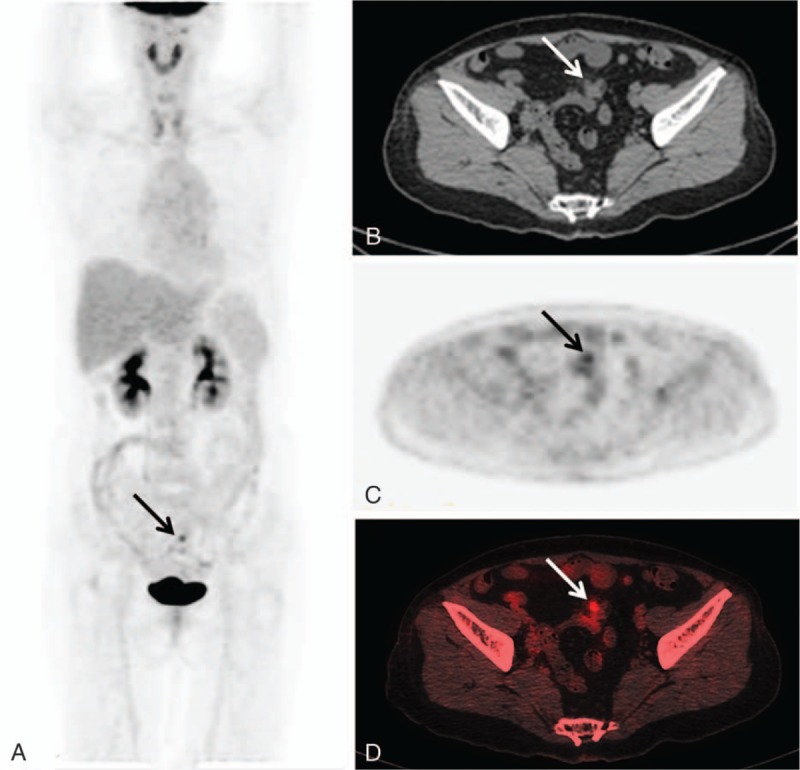
The maximum intensity projection PET image of ^18^F-FDG PET/CT scan (A) revealed a focal increased ^18^F-FDG uptake lesion (arrow) and normal ^18^F-FDG uptake of the pancreas. Transverse CT (B), and corresponding PET (C) and fusion (D) images showed the lesion (arrow) with the SUV_max_ of 4.3 in the small intestine. CT = computed tomography,^18^F-FDG PET = ^18^F-fluorodeoxyglucose positron-emission tomography, SUV_max_ = maximum standardized uptake value.

**Figure 2:**
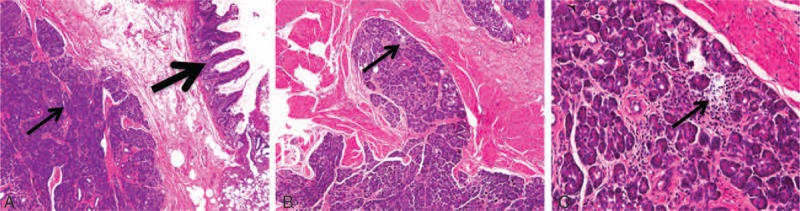
Low-magnification images of ^18^F-FDG PET/CT scan (A and B, hematoxylin-eosin [HE] × 40) demonstrated the normal small intestine mucosa (thick arrow) and lobules of heterotopic pancreatic acini (thin arrows) in the submucosa. High-magnification image (C, HE × 200) of image F revealed destruction of the acini with infiltration of lymphocytes (thin arrow). CT = computed tomography,^ 18^F-FDG PET = ^18^F-fluorodeoxyglucose positron-emission tomography, HE = hematoxylin-eosin.

A 60-year-old woman was referred to our hospital due to a 10-day history of abdominal pain with elevated levels of serum amylase (431 U/L; reference range, 0–108 U/L) and lipase (627 U/L; reference range, 23–300 U/L). Abdominal CT and ultrasound examinations were negative. In order to search for the cause, an ^18^F-FDG PET/CT whole body scan was performed after the injection of 222 MBq (7 mCi) of ^18^F-FDG with a blood glucose level of 5.3 mmol/L. The maximum intensity projection PET image (Fig. 1A) revealed a focal increased ^18^F-FDG uptake lesion (arrow) and normal ^18^F-FDG uptake of the pancreas. Transverse CT (Fig. 1B), and corresponding PET (Fig. 1C) and fusion (Fig. 1D) images showed the lesion (thin arrow) with the SUV_max_ (maximum standardized uptake value) of 4.3 in the small intestine. Then, complete resection of the lesion was performed and abdominal pain disappeared. Low-magnification images (Fig. 2A and B, hematoxylin-eosin [HE] × 40) demonstrated the normal small intestine mucosa (thick arrow) and lobules of heterotopic pancreatic acini (thin arrows) in the submucosa. High-magnification image (Fig. 2C, HE × 200) of image F revealed destruction of the acini with infiltration of lymphocytes, indicating active chronic inflammation (arrow). The findings are consistent with a diagnosis of intestinal heterotypic pancreatitis.

A written informed consent for the case report was obtained from the patient. The consent procedure was approved by the Ethics Committee of Shanghai Jiao Tong University Affiliated Sixth People's Hospital.

## 3 Discussion

HP can induce complications including inflammation, ulceration, chemical irritation, bleeding, obstruction, malignant transformation, jejunal intussusception, and ileus.^[6–10]^ Surgical excision is the first and best choice of treatment because medical treatment is not effective.^[6,10]^ However, the preoperative diagnosis of HP in the small intestine is difficult. Symptoms depend on the size of lesion and involvement of mucosa.^[10]^ HP can frequently be mistaken as gastrointestinal stromal tumor or leiomyoma at endoscopy, ultrasonography, or CT scanning.^[10,11]^ To our knowledge, HP with increased ^18^F-FDG accumulation has only been reported in 2 reports with the lesions in the stomach with the SUV_max_ of 4.0^[12]^ and esophagus with the SUV_max_ of 10.0, which was concerned for a neoplasm before surgery.^[13]^ However, the lesion with increased ^18^F-FDG accumulation in small intestine has not been reported before. As the inflammatory behavior of HP is similar to acute pancreatitis or focal exacerbation of chronic pancreatitis which occurs in the normal pancreatic gland,^[14–18]^ increased ^18^F-FDG uptake in HP can be explained.^[19–23]^ Notably, a high glucose metabolic activity in pancreatic tissues cannot distinguish neoplasm from inflammation.^[24]^ The PET/CT finding with the noted ^18^F-FDG uptake in this case likely represented a localized inflammatory process, in accordance with the patient's symptomatology and the relatively low SUV_max_ of 4.3.

In conclusion, this case indicated that heterotopic pancreatitis in small intestine with focal ^18^F-FDG uptake should be considered when differing from leiomyoma,^[25]^ lymphoma,^[26]^ gastrointestinal stromal tumor,^[27]^ and intestinal tuberculosis.^[28]^
